# Mechanisms Limiting Ti_3_SiC_2_ Formation During Carbothermal Reduction of Liquid Ti-Si-C-O Precursors

**DOI:** 10.3390/ma19163416

**Published:** 2026-08-12

**Authors:** Yuanjie Wang, Wenqian Wang, Zilei Chen, Zongwei Guo, Liyan Chen, Yue Yang, Yuhui Ao

**Affiliations:** 1 School of Materials Science & Engineering, Zhejiang Sci-Tech University, Hangzhou 310018, China; 2 Innovation Center of Advanced Textile Technology (Jianhu Laboratory), Shaoxing 312030, China

**Keywords:** Ti_3_SiC_2_, carbothermal reduction, precursor-derived ceramics, phase evolution

## Abstract

The traditional precursor-derived ceramic (PDC) route uses micron-sized titanium sources and silicon carbide precursors to prepare Ti_3_SiC_2_, which limits its application in fine-scale fields. We propose a fully liquid Ti-Si-C-O precursor, consisting of tetrabutyl titanate (TBT) and liquid polycarbosilane (LPCS), inspired by the carbothermal reduction of solid oxides to prepare Ti_3_SiC_2_. The fully liquid precursor can offer processing advantages that are not achievable through previous PDC processes. The pyrolysis of the TSO-1 (TBT:LPCS = 3:2) undergoes four stages: dehydration condensation (RT~200 °C), alkoxy removal (200~400 °C), inorganic conversion (400~800 °C), and carbothermal reduction (>800 °C). The products of TSO-1 pyrolyzed to 1400 °C and 1600 °C are Ti_3_O_5_, SiO_2_ and TiC, and Ti_3_O_5_, Ti_2_O_3_, SiO_2_ and TiC, respectively. Increasing the LPCS content in the system can promote the formation of TiC, but will not produce Ti_3_SiC_2_. This phenomenon stems from the dual constraints: thermodynamically, titanium oxides outcompete SiO_2_ for carbon; kinetically, the gaseous escape of effective carbon and its microscale non-uniform distribution further impair the carbothermal reduction of SiO_2_. These combined factors prevent the gas–solid reaction from establishing, rendering Ti_3_SiC_2_ unattainable.

## 1. Introduction

Ti_3_SiC_2_ is a typical MAX phase, which combines the characteristics of metal and ceramic, including excellent electrical conductivity, thermal conductivity, machinability, oxidation resistance and damage tolerance [1,2,3,4]. These comprehensive properties give it broad application prospects in the fields of high temperature structural materials, electrical contact materials, lubricating materials, composite interface layers, protective coatings and so on [5,6].

At present, a variety of routes have been developed for the preparation of Ti_3_SiC_2_, including pressureless sintering, hot pressed sintering, hot isostatic pressing, spark plasma sintering, microwave sintering, reactive melt infiltration, molten salt synthesis, chemical vapor deposition and precursor-derived ceramic (PDC) [7,8,9,10,11,12,13,14]. Among them, the PDC route offers advantages in processability and formability of organic precursors. It can achieve the near-net-shape forming of target ceramic materials through impregnation, cross-linking and high-temperature pyrolysis, especially in the preparation of complex structural components [15]. However, most of the Ti_3_SiC_2_ precursor systems in the existing literature are physically blended with micron-sized titanium sources (Ti or TiH_2_) and silicon carbide precursors [14,16,17,18,19,20]. The resulting target products are mostly powders or bulk ceramics, and slurries composed of micron-sized particles cannot readily meet the requirements of fine-scale applications such as thin interface layers.

The carbothermal reduction of oxide mixtures is another important solid-state synthesis route for the preparation of Ti_3_SiC_2_. For example, Goldin et al. [21] obtained a material containing 60 wt% Ti_3_SiC_2_ through vacuum carbothermal reduction, using Yaregskoe leucoxene concentrate containing about 50 wt% TiO_2_ and 40 wt% SiO_2_. Cetinkayan and Eroglu et al. [22,23] prepared Ti_3_SiC_2_ by carbothermal reduction of the mixture of TiO_2_ and SiO_2_ in an argon atmosphere, but the product yield did not exceed 40 wt%. The study found that the formation of Ti_3_SiC_2_ by carbothermal reduction is strongly dependent on the composition of the reactants and their contact states.

Inspired by the above carbothermal reduction route, and in order to overcome the limitations of micron-scale slurries in the traditional PDC route, this work proposes a fully liquid Ti-Si-C-O precursor route, using tetrabutyl titanate (TBT) as the titanium dioxide precursor and liquid hyperbranched polycarbosilane (LPCS) as the silica and carbon precursors. Both components are liquid, which can achieve uniform mixing at the molecular level. The liquid Ti-Si-C-O precursor can be applied to processes such as flow-casting, impregnation, and spraying. In this study, we systematically investigated the pyrolysis behavior of the precursor and the phase evolution of the carbothermal reduction products, aiming to reveal the key limits in the formation of Ti_3_SiC_2_ and its internal mechanism.

## 2. Materials and Methods

Liquid polycarbosilane (LPCS) with an approximate constitutional formula of [SiH_1.9_(CH_2_CH=CH_2_)_0.1_CH_2_]_n_ was purchased from Zhengzhou Alpha Chemical Co., Ltd. (Zhengzhou, China). The weight-average molecular weight (M_w_) was 6273 g/mol, and the number-average molecular weight (M_n_) was 1176 g/mol. The elemental composition and empirical formula of LPCS are listed in Table 1. Tetrabutyl titanate (TBT) and xylene were purchased from Shanghai Maclin Biochemical Technology Co., Ltd. (Shanghai, China). Xylene was dried with 3Å molecular sieve before use.

Different proportions of LPCS and TBT mixtures were prepared, and their denotations and compositions are listed in Table 2. The LPCS and TBT were also studied for comparison and were used as reference materials.

Firstly, the LPCS and TBT were uniformly dispersed in a xylene solution to form a stable suspension. Then, xylene was removed from the suspension by vacuum distillation to obtain a uniform mixture.

**Figure 1:**
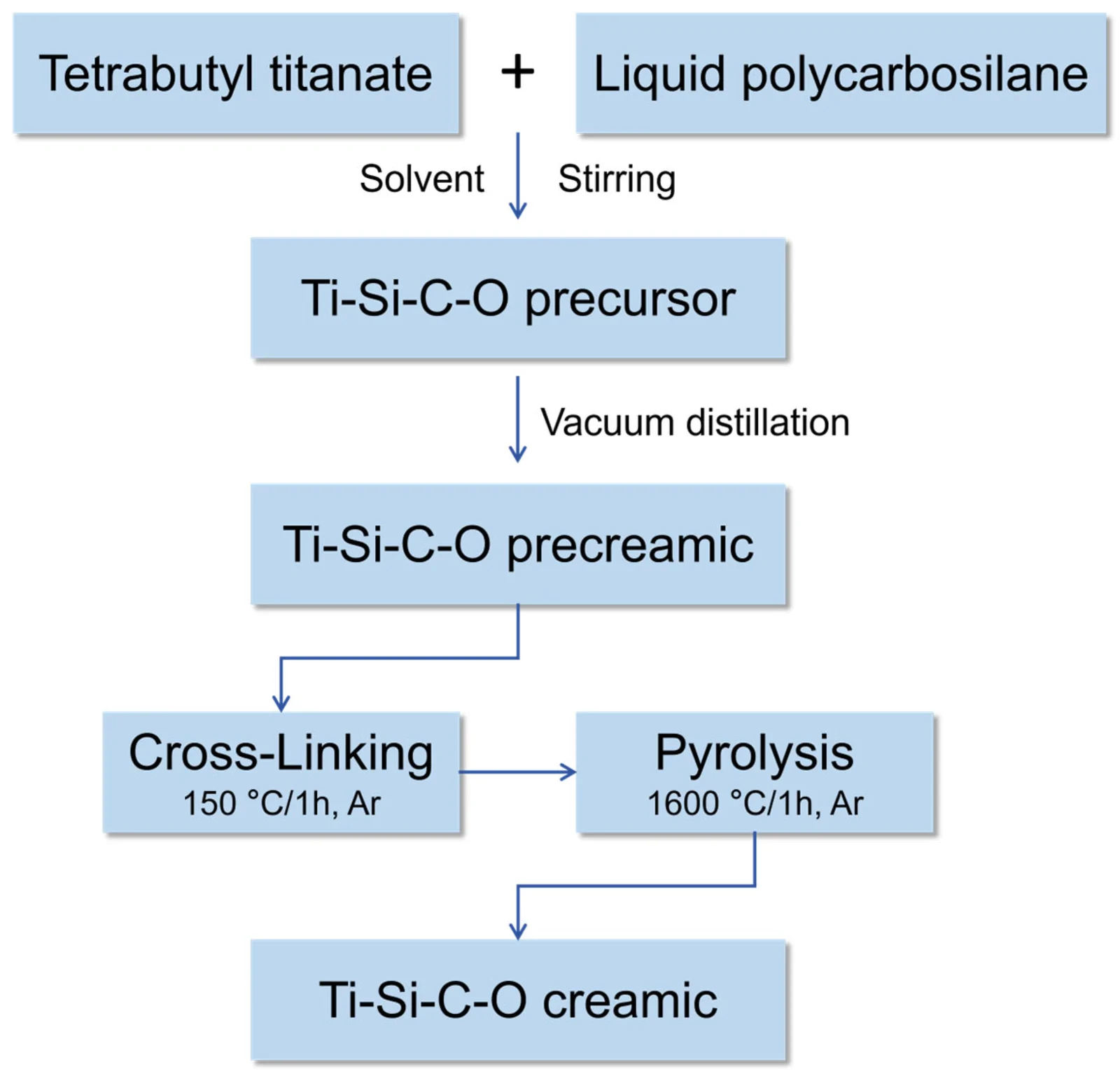
Flow chart from Ti-Si-C-O precursor to final precursor-derived ceramics.

The above mixture was put in an alumina crucible and cured in a furnace at 150 °C under an argon atmosphere for 1 h. Afterwards, the cured solid mixture was transferred to a graphite crucible for further pyrolysis at temperatures from 200 °C to 1600 °C under an argon atmosphere, with a holding time of 1 h at each heat-treatment temperature (Figure 1).

The contents of elements C, H and O were determined by elemental analyzer (UNICUBE^®^, Elementar Analysensysteme, Hanau, Germany). The Si content was calculated by difference. Thermogravimetry (TG) analysis was conducted on the thermal analyzer (SDT 650, TA Instruments, New Castle, DE, USA). During the thermal weight loss test, the temperature was raised to 1350 °C at a rate of 10 °C/min in an Ar atmosphere. Mass spectrometry (MS) analysis of volatile gas compounds produced during the TG test was conducted on the MS mass spectrometer (Discovery, TA Instruments, New Castle, DE, USA). Fourier transform infrared (FTIR) spectra were recorded on infrared spectrometer (INVENIO R, Bruker Corporation, Rheinstetten, Germany). The phase composition was determined by X-ray diffractometer (Empyrean, Malvern Panalytical, Almelo, The Netherlands) (40 kV/40 mA, Cu-K*_α_*). The scanning speed was 10 °/min and the scanning angles ranged from 20° to 80°. According to the XRD pattern, the mass percentages of the phases were calculated using the RIR value method. Raman scattering spectra measurements were conducted with a raman spectrometer (HORIBA Scientific, LabRAM HR Evolution, Paris, France), using a laser with wavelength of 532 nm. The morphology of the samples was characterized by scanning electron microscope (ZEISS, Sigma 500, Oberkochen, Germany) at 7 kV acceleration voltage. Energy dispersive spectrometer (EDS) analysis was conducted to differentiate the phases. In order to ensure the reproducibility of the measurements, all experiments were repeated multiple times. The data presented in this paper are representative results.

## 3. Results and Discussion

### 3.1. Pyrolysis Behavior of the TSO-1

As reported by Cetinkaya et al., Ti_3_SiC_2_ was synthesized via the carbothermal reduction method using TiO_2_, SiO_2_ and C as raw materials, with the overall reaction expressed in Equation (1). Their results indicated that the reaction between solid Ti_2_O_3_ and gaseous SiO/CO played a dominant role in the formation of Ti_3_SiC_2_ (Equations (2)–(6)) [23].

3 TiO_2_(s) + 2 SiO_2_(s) + 11 C(s) → Ti_3_SiC_2_(s) + SiO(g) + 9 CO(g) (1)

n TiO_2_(s) + C(s) → Ti_n_O_2n−1_(s) + CO(g) (2)

SiO_2_(s) + C(s) → SiO(g) + CO(g) (3)

3 Ti_2_O_3_(s) + 2 SiO(g) + 19/2 C(s) → 2 Ti_3_SiC_2_(s) + 11/2 CO_2_(g) (4)

3 Ti_2_O_3_(s) + 2 SiO(g) + 15 C(s) → 2Ti_3_SiC_2_(s) + 11 CO(g) (5)

3 Ti_2_O_3_(s) + 2 SiO(g) + 19 CO(g) → 2Ti_3_SiC_2_(s) + 15 CO_2_(g) (6)

According to the molar ratio of raw materials (TiO_2_:SiO_2_:C = 3:2:11) in Equation (1), the ratio of TBT to LPCS was calculated. Assuming that TBT can be converted into TiO_2_ and LPCS can be converted into SiO_2_ and carbon during pyrolysis, the ideal molar ratio of TBT to LPCS is 3:2, and this composition is denoted as TSO-1. The obtained TSO-1 precursor can remain in a liquid state for approximately 6 h under sealed conditions at room temperature, and then gradually gel and form a light-yellow solid.

**Figure 2:**
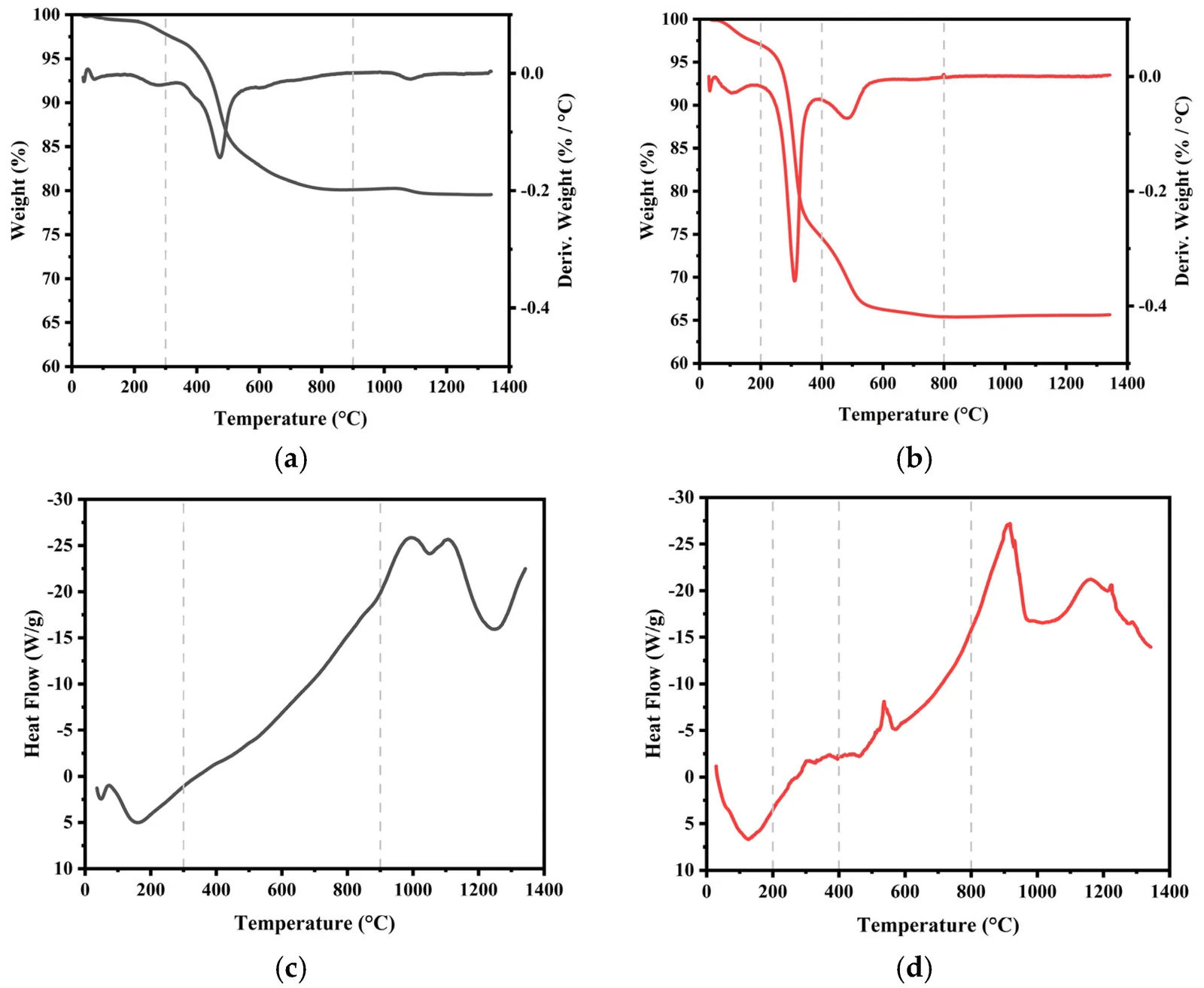
TG and DSC curves of (**a**,**c**) LPCS and (**b**,**d**) TSO-1.

In order to clarify the pyrolysis behavior of TSO-1, the 150 °C-cured products of LPCS and TSO-1 were examined by TG-DSC-MS. Figure 2 shows the TG and DSC curves of LPCS and TSO-1. The ceramic yields of LPCS and TSO-1 at 1350 °C are 79.5 wt% and 65.6 wt%, respectively.

According to the DTG curve, the pyrolysis process of LPCS can be divided into three stages [24]: (1) In the first stage (room temperature ~300 °C), the weight loss is about 2.2 wt%, and the DSC curve exhibits a slight endothermic process, which mainly corresponds to the volatilization of low-molecular-weight LPCS prior to complete crosslinking. (2) In the second stage (300~900 °C), the weight loss is about 17.7 wt%, mainly attributed to the condensation of groups and the detachment of side groups during the inorganic transformation of LPCS. (3) In the third stage (900~1350 °C), the weight loss is about 0.6 wt%. The DSC curve shows an exothermic peak at 1000 °C and a slight endothermic process at 1200~1300 °C, which are attributed to the structural relaxation/condensation of amorphous SiC before crystallization and the decomposition of SiO_x_C_y_, respectively.

**Figure 3:**
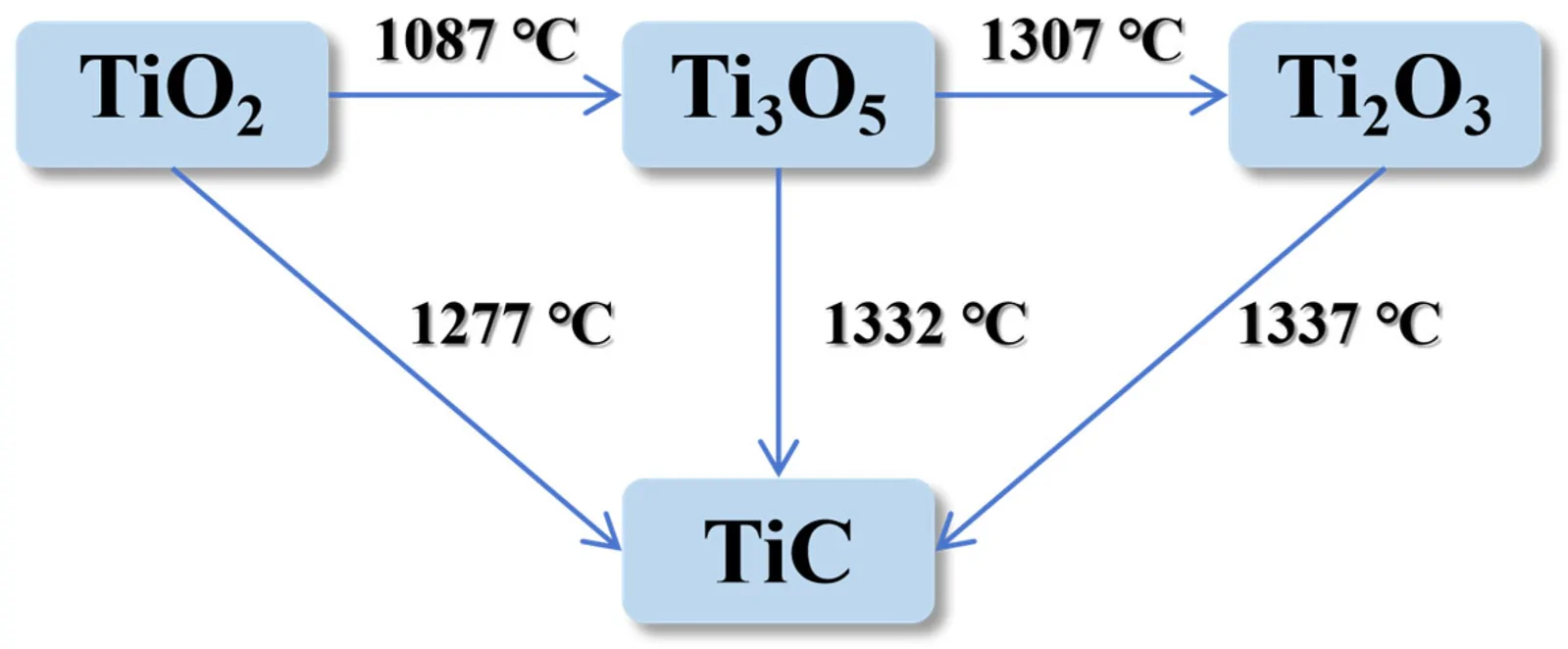
The carbothermal reduction pathway of TiO_2_.

Compared with LPCS, the pyrolysis behavior of TSO-1 is markedly different. According to the DTG curve, the pyrolysis process of TSO-1 can be divided into four stages: (1) In the first stage (room temperature ~200 °C), the weight loss is about 3.0 wt%, and the DSC curve shows an obvious endothermic process, which is presumed to arise from the dehydration condensation of hydroxyl groups in TSO-1. (2) In the second stage (200~400 °C), the weight loss is about 17.3 wt%, which may be caused by the pyrolysis and volatilization of the TBT component in TSO-1 (the boiling point of TBT is about 310 °C) [25]. (3) In the third stage (400~800 °C), the weight loss is about 14.3 wt%, attributed to the condensation and shedding of Si-CH_x_ groups in TSO-1. The DSC curve shows an exothermic peak at 535 °C, which is ascribed to the transformation of anatase to rutile phase and the crystallization of the rutile phase [26]. (4) In the fourth stage (800~1350 °C), the residual mass is basically unchanged, and the DSC curves exhibit obvious endothermic peaks at 900~1200 °C and 1200~1350 °C, which may correspond to the carbothermal reduction of TiO_2_ and Ti_3_O_5_, respectively (Figure 3).

**Figure 4:**
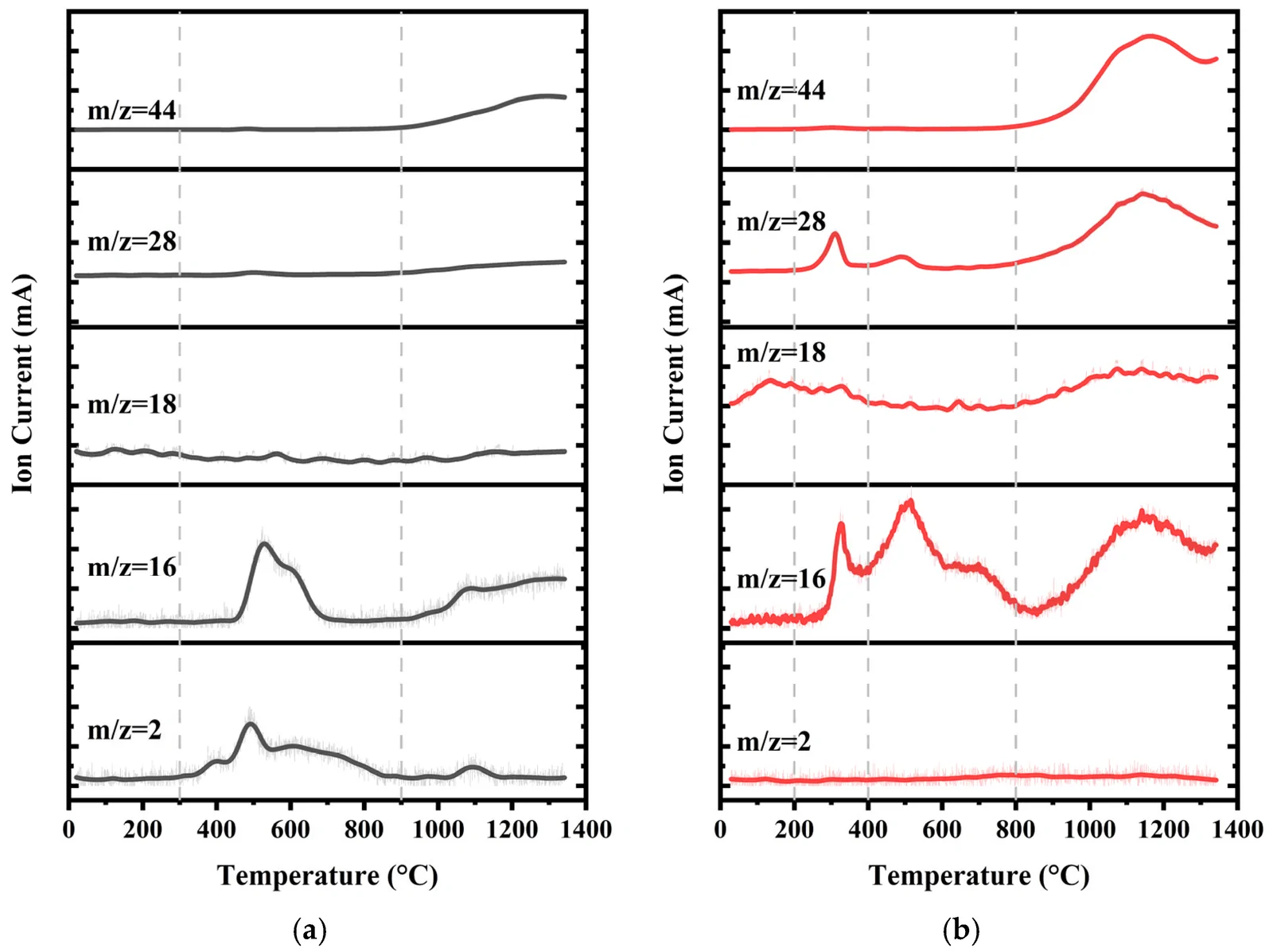
MS spectrum of the gas released by (**a**) LPCS and (**b**) TSO-1 during pyrolysis.

The mass spectrometry data were further analyzed, and five types of fragment ions with relatively high intensities during pyrolysis were selected for assignment. The corresponding functional groups are listed in Table 3. The MS spectrum of the gases released from LPCS and TSO-1 during pyrolysis are shown in Figure 4.

For pure LPCS, a release peak of H_2_ is observed in the range of 300~900 °C, and CH_4_ shows a significant release peak in the range of 400~700 °C, which corresponds to the group condensation and side-group shedding in the inorganic transformation of LPCS. At about 1100 °C, weak release peaks of H_2_ and CH_4_ appear, corresponding to the further condensation of amorphous SiC before crystallization. In the range of 1100~1350 °C, continuous weak release of O^+^, CO and SiO is detected, corresponding to the decomposition of SiO_x_C_y_.

Compared with LPCS, TSO-1 exhibits H_2_O release throughout the entire test range, whereas H_2_ release is virtually absent. TSO-1 undergoes hydrolysis to form a large number of hydroxyl groups, which preferentially undergo dehydration condensation reactions, releasing H_2_O at lower temperatures. Meanwhile, the carbothermal reduction reaction at elevated temperatures creates a strongly oxygen-consuming environment. Even if a small amount of H_2_ is produced, it can be captured and oxidized to H_2_O, resulting in the detection of H_2_O at high temperatures. In the range of 200~400 °C, obvious release peaks of hydrocarbons (CH_4_ and C_2_H_4_) are observed, which are consistent with the thermal decomposition of alkoxy groups in TSO-1. In the range of 1100~1350 °C, the signals of O^+^, CO and SiO are significantly enhanced, which may be associated with the carbothermal reduction of TSO-1.

**Figure 5:**
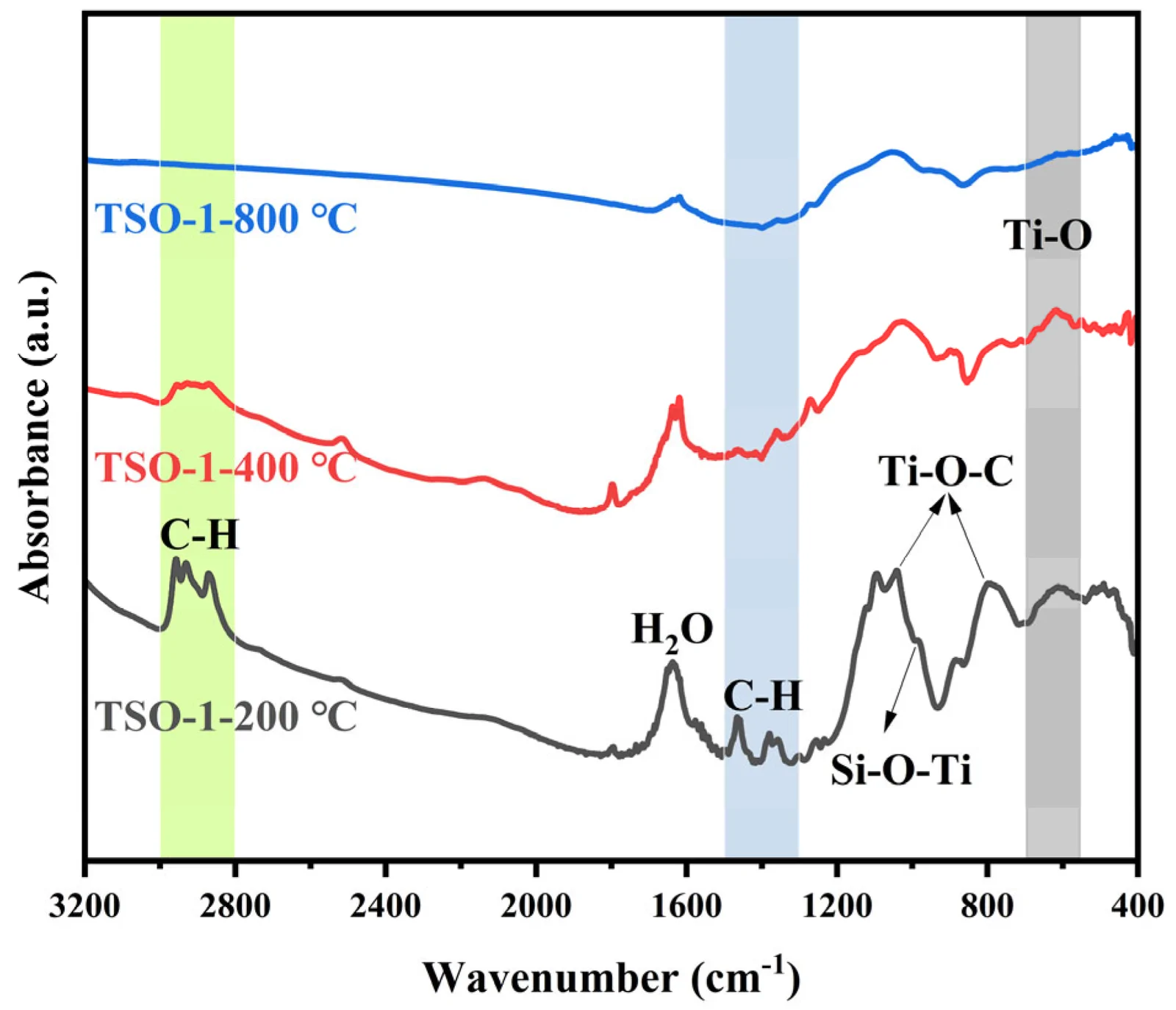
FT-IR spectra of the pyrolysis products of TSO-1 at different temperatures.

Based on the TG-DSC-MS results, TSO-1 was pyrolyzed at 200 °C, 400 °C and 800 °C, respectively, and the products were characterized by FT-IR. Figure 5 shows the FT-IR spectra of the pyrolysis products of TSO-1 at different temperatures, and Table 4 lists the characteristic infrared absorption peaks corresponding to selected functional groups.

**Figure 6:**
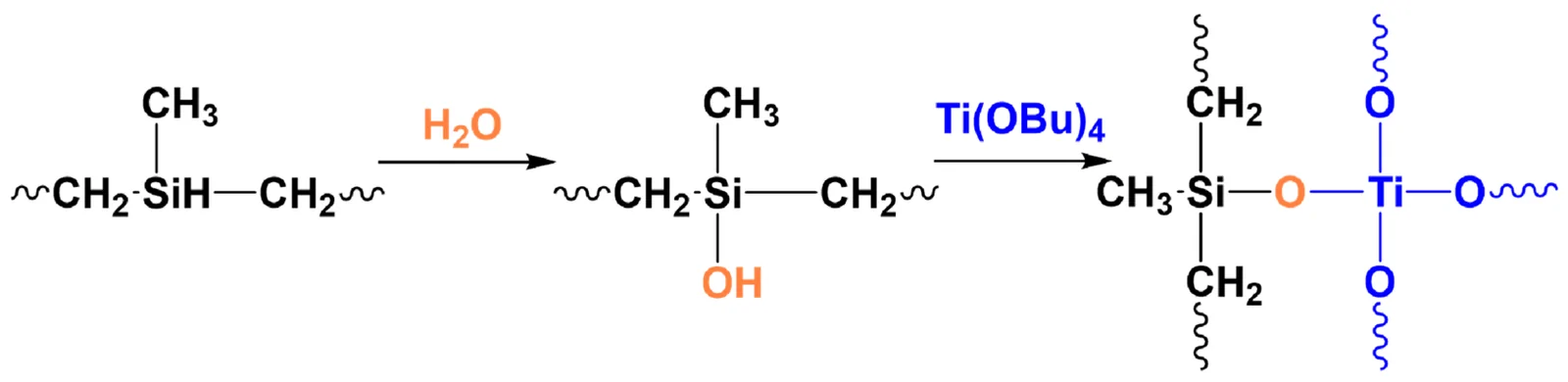
The schematic diagram of the dehydration condensation reaction between Ti-OH and Si-OH.

As shown in Figure 5, when the pyrolysis temperature reaches 200 °C, the FT-IR spectrum of TSO-1 mainly exhibits C-H, Ti-O-C and Si-O-Ti absorption peaks. The appearance of Si-O-Ti absorption peak indicates that a dehydration condensation reaction has occurred between Ti-OH and Si-OH (Figure 6) [27]. When the pyrolysis temperature reached 400 °C, the intensity of the C-H absorption peaks decreased, indicating that TSO-1 had undergone dealkylation reaction, which is consistent with the rapid weight loss observed in the second stage of thermal analysis (200~400 °C) and the release of hydrocarbons (CH_4_, C_2_H_4_) detected by MS. Simultaneously, the Ti-O-C absorption peak weakens, while the Ti-O vibration peak begins to intensify, indicating that the Ti-O-C bonds are converted into the Ti-O-Ti network structure, as the TiO_2_ framework begins to form. When the pyrolysis temperature reached 800 °C, the C-H absorption peaks disappeared completely, indicating that all organic groups have been removed, and the TSO-1 precursor has completed the transition from organic to inorganic state. Moreover, the Ti-O-C absorption peaks are also largely eliminated, and the Ti-O vibration peak is further broadened and strengthened, indicating that the TiO_2_ inorganic network is fully established.

### 3.2. Phase Evolution of the TSO-1

**Figure 7:**
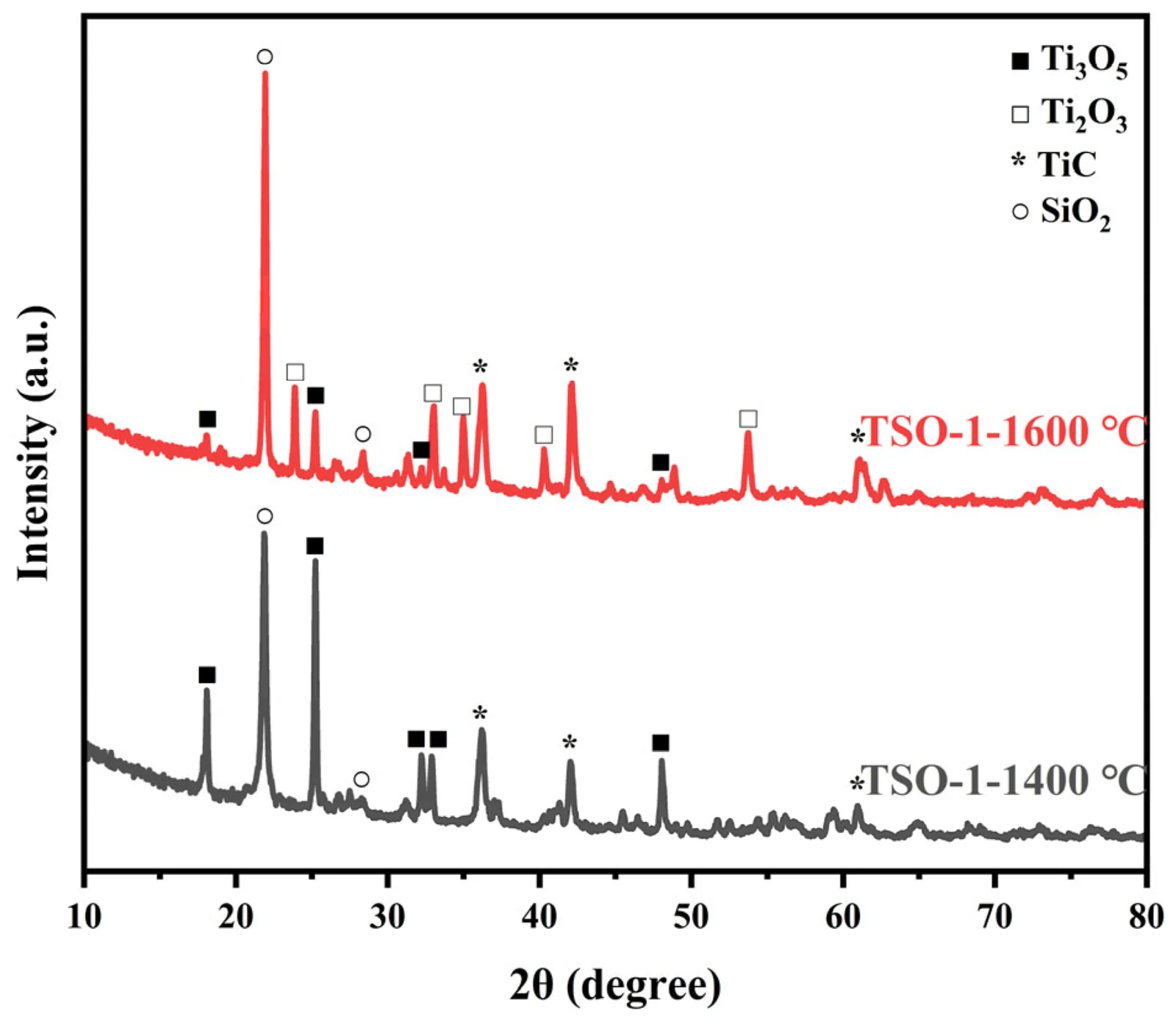
XRD patterns of TSO-1 products sintered at 1400 °C and 1600 °C.

Figure 7 presents the XRD patterns of TSO-1 products sintered at 1400 °C and 1600 °C, and Table 5 lists the quantitative phase mass percentages. At 1400 °C, the pyrolyzed product of TSO-1 contains three phases: Ti_3_O_5_ (PDF#82-1137), SiO_2_ (PDF#82-0512) and TiC (PDF#65-8805). The absence of TiO_2_ in the product indicates that the carbothermal reduction reaction of TiO_2_ to Ti_3_O_5_ has been completed during the pyrolysis process. This observation is consistent with the endothermic peak at 900~1200 °C in the TG-DSC curve (corresponding to the carbothermal reduction of TiO_2_). There is no Ti_2_O_3_ phase in the product, suggesting that the TiC phase is derived directly from the carbothermal reduction reaction of TiO_2_. At 1600 °C, the mass percentage of Ti_3_O_5_ phase decreases significantly, the Ti_2_O_3_ phase (PDF#89-4746) appears, and the mass percentage of the TiC and SiO_2_ phases increase. The increased SiO_2_ mass percentage may be due to the continuous escape of CO gas, which reduces the total mass of the sample, and thus passively raises the relative proportion of SiO_2_ in the pyrolysis products.

**Figure 8:**
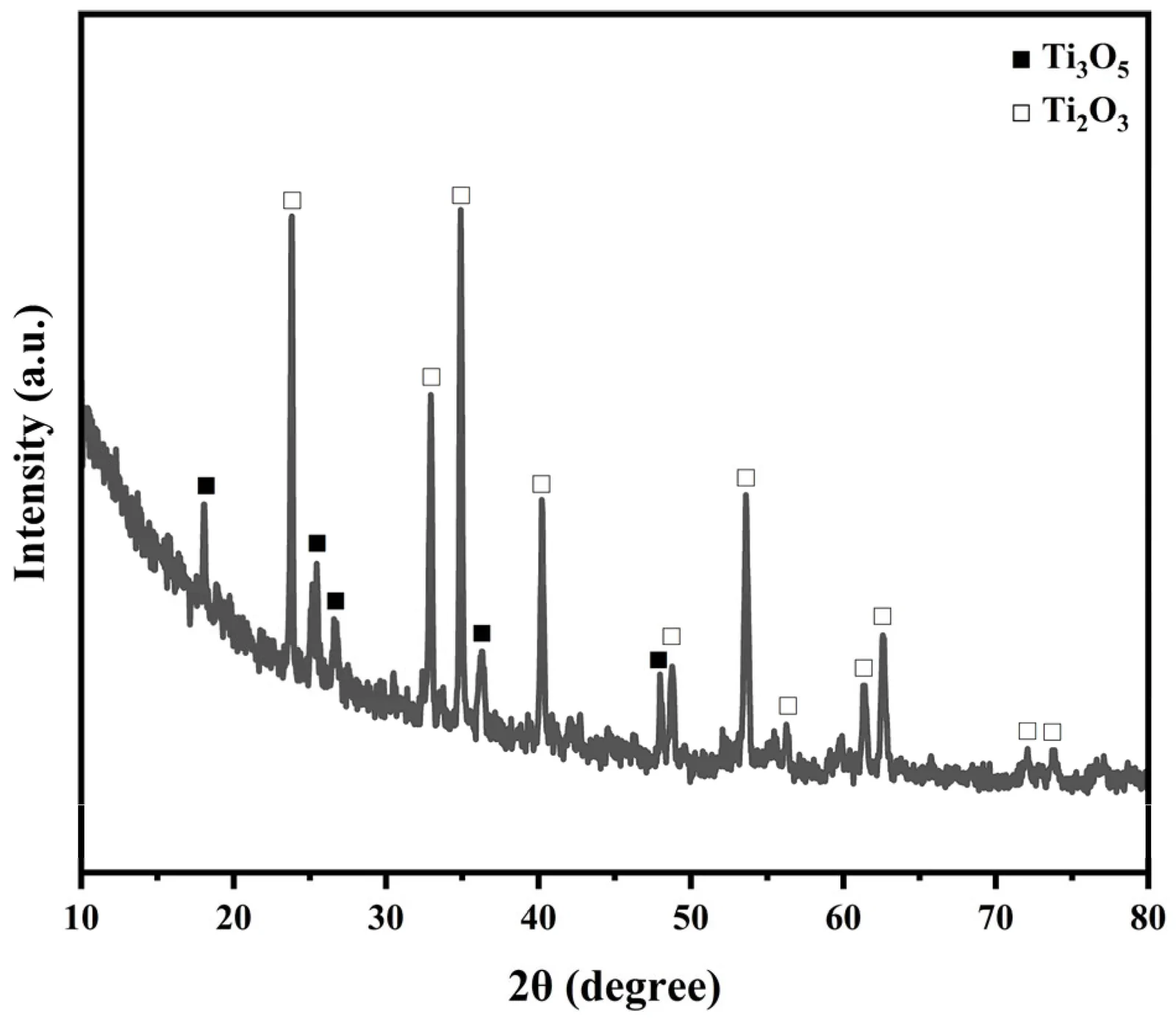
XRD patterns of TBT sintered at 1600 °C.

In order to establish the reference baseline for the carbothermal reduction capability of the titanium source alone, the phase composition of TBT sintered at 1600 °C was analyzed. Figure 8 shows the XRD pattern of TBT sintered at 1600 °C. The pattern reveals that the pyrolysis products of TBT are mainly Ti_2_O_3_ and Ti_3_O_5_. This indicates that the amount of carbon produced by the decomposition of butoxy groups (-OC_4_H_9_) in the TBT molecule is extremely limited, such that TiO_2_ can only be reduced to the Ti_3_O_5_ and Ti_2_O_3_ stages and cannot be further reduced to TiC. In contrast to TBT, the additional carbon source provided by LPCS in TSO-1 enables further carbothermal reduction of Ti_2_O_3_ to the TiC stage. Although substantial SiO and CO gases were detected during the pyrolysis of TSO-1, Ti_3_SiC_2_ was not observed in the product. This fact indicates that the partial pressure and proportion of SiO and CO in TSO-1 may not satisfy the conditions required for the Ti_3_SiC_2_ gas–solid reaction path (Equations (4)–(6)). Furthermore, owing to the insufficient effective carbon content, SiO_2_ cannot be fully reduced to SiO and CO, resulting in interruption of the gas–solid reaction due to a lack of sufficient gaseous reactants.

**Figure 9:**
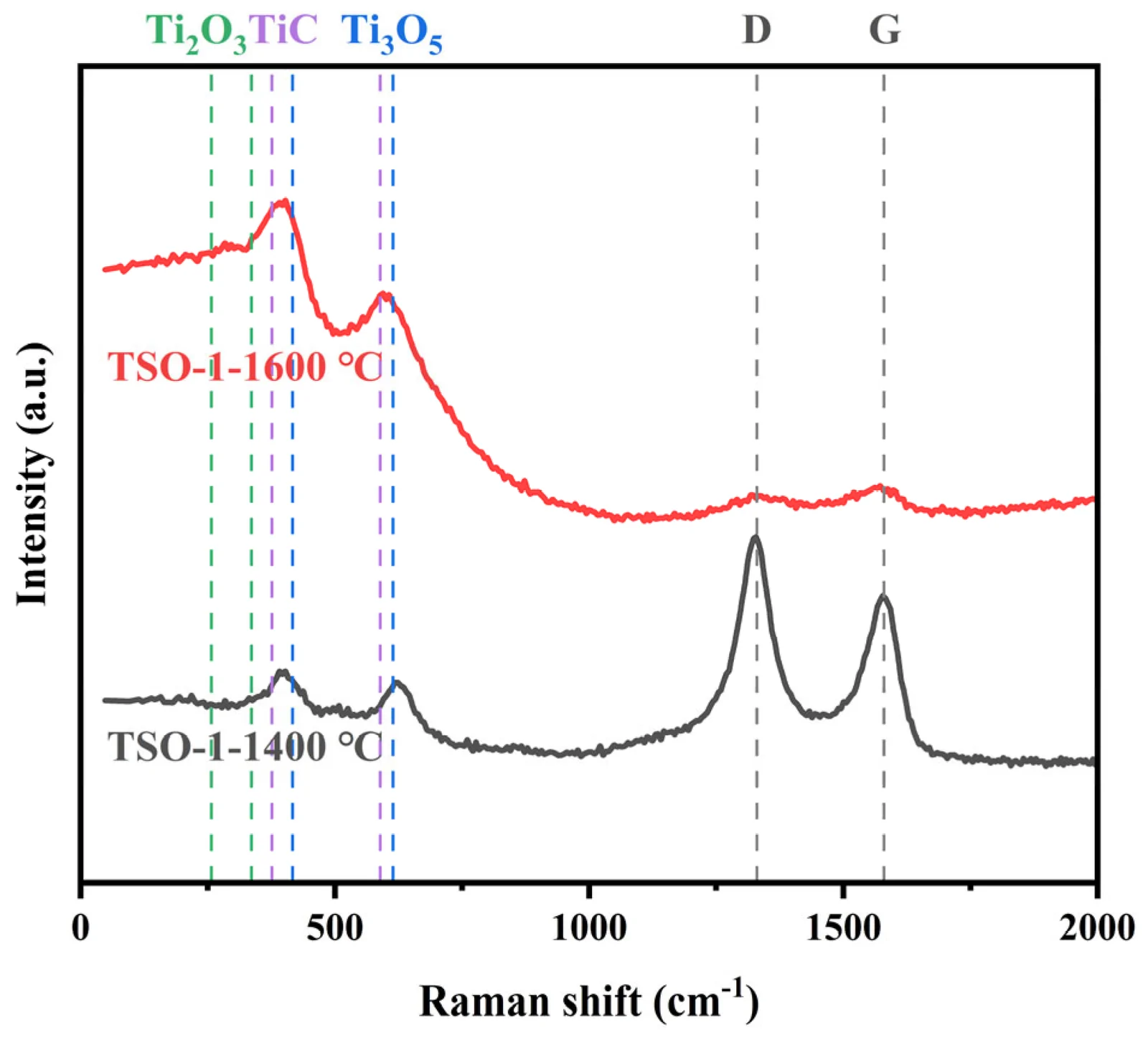
Raman spectra of TSO-1 sintered at 1400 °C and 1600 °C.

Figure 9 shows the Raman spectra of TSO-1 sintered at 1400 °C and 1600 °C. The Raman spectra of different sintering temperatures have peaks corresponding to Ti_3_O_5_ and free carbon at 417 cm^−1^, 614 cm^−1^, 1330 cm^−1^ and 1580 cm^−1^ [28]. Compared with the products sintered at 1400 °C, the intensity of D peak and G peak associated with free carbon in the spectrum of products sintered at 1600 °C decreased significantly, with the I_D_/I_G_ ratio decreasing from 1.4 to 0.8. The weak signal peak attributed to Ti_2_O_3_ began to appear at 257 cm^−1^, while the weak signal peaks attributed to TiC emerged at 284 cm^−1^, 376 cm^−1^, 589 cm^−1^ and 668 cm^−1^. This indicates that with the increase in sintering temperature, Ti_3_O_5_ gradually consumes free carbon to reduce to Ti_2_O_3_ and TiC, and the graphitization degree of free carbon is also improved [29].

**Figure 10:**
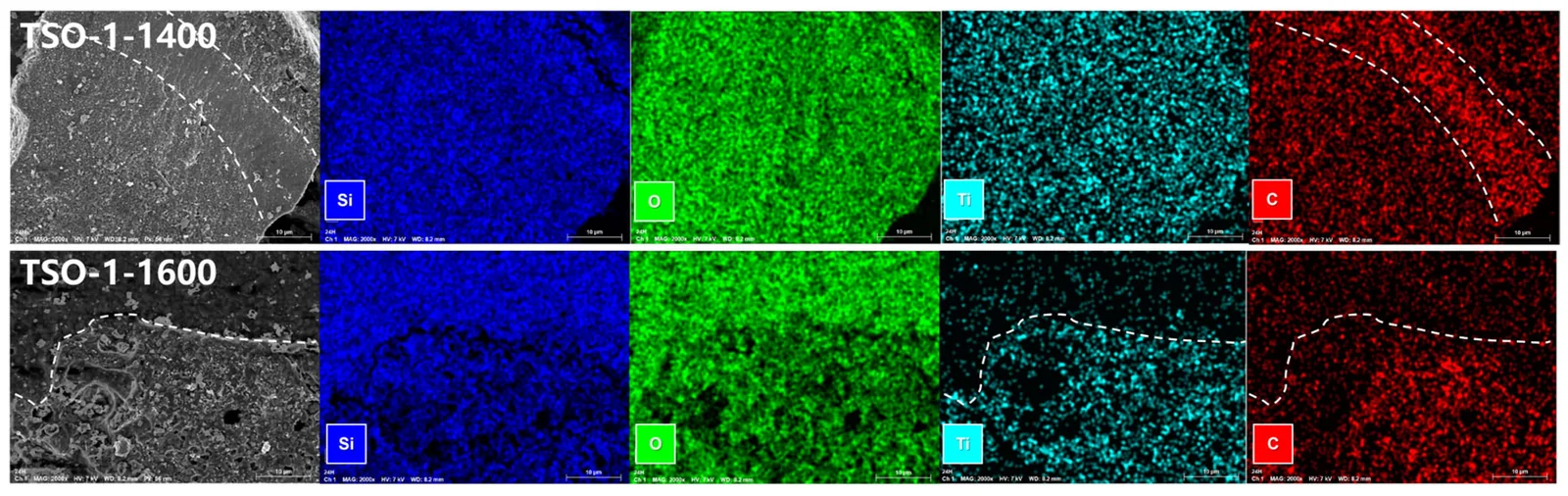
SEM images and the EDS mapping analysis of TSO-1 sintered at 1400 °C and 1600 °C.

Figure 10 shows the SEM images and the EDS mapping analysis of TSO-1 sintered at 1400 °C and 1600 °C. The results of EDS map scanning showed that the three elements of Ti, Si and O in the product sintered at 1400 °C are evenly distributed in the observation area, while the C element exhibits localized band-like enrichment. From the morphological perspective, the band-like enrichment area of C element is relatively dense, and there are only a small number of micropores formed by the escape of pyrolysis gas in other areas. The band-like enrichment of C element may be derived from the micro-phase separation during the gelation process of the precursor. The LPCS-enriched region forms a relatively dense enrichment region of C element due to the higher carbon yield in the subsequent pyrolysis. The region of Si, O enrichment and Ti, C depletion appears in the product sintered at 1600 °C, which is relatively dense and has coarse particles. It is likely that at 1600 °C, the carbothermal reduction reaction becomes more intense, and the titanium oxide is preferentially reduced and migrated in the enrichment region of C element, resulting in the simultaneous consumption of Ti and C in the local region. Meanwhile, the unreduced SiO_2_ phase undergoes sintering at 1600 °C, achieving densification and grain growth through viscous flow and diffusion mass transfer, and finally a dense region rich in Si-O is formed. Ti, Si, O and C are uniformly distributed in the other regions of the product sintered at 1600 °C, and there are a large number of pores. Due to the low carbon content in the other regions, the carbothermal reduction reaction is carried out in a continuous and mild manner, and the gas escapes evenly to form a large number of pores. The consumption rate of each element is relatively matched, so there is no obvious segregation.

Based on the results of TG-DSC-MS, FT-IR, XRD, Raman and SEM analysis, the pyrolysis and carbothermal reduction process of the TSO-1 precursor can be summarized as follows: From room temperature to 200 °C, the dehydration condensation stage occurs, during which TBT and LPCS are chemically connected via Si-O-Ti bonds, accompanied by a weight loss of about 3.0 wt%. At 200~400 °C, the alkoxy groups are removed, and the organic groups escape in the form of hydrocarbons, with a weight loss of about 17.3 wt%, during which TiO_2_ begins to form. At 400~800 °C, the inorganic conversion stage takes place, with a weight loss of 14.3 wt%. At 800~1350 °C, the carbothermal reduction stage commences, the residual mass remains basically unchanged, and TiO_2_ is gradually reduced to titanium suboxides. The products of TSO-1 sintered at 1400 °C are Ti_3_O_5_, SiO_2_ and TiC. At 1600 °C, Ti_3_O_5_ was partially converted into Ti_2_O_3_ and TiC, SiO_2_ persists, and Ti_3_SiC_2_ is not detected. The above results are attributed to the large amount of escape of organic carbon during the pyrolysis of TSO-1, which leads to the insufficient total amount of effective carbon in the system, while the non-uniform distribution of residual carbon and the partitioned carbothermal reduction pathway further inhibit the gas–solid reaction path of Ti_3_SiC_2_.

### 3.3. Effect of Precursor Composition on Phase Composition

**Figure 11:**
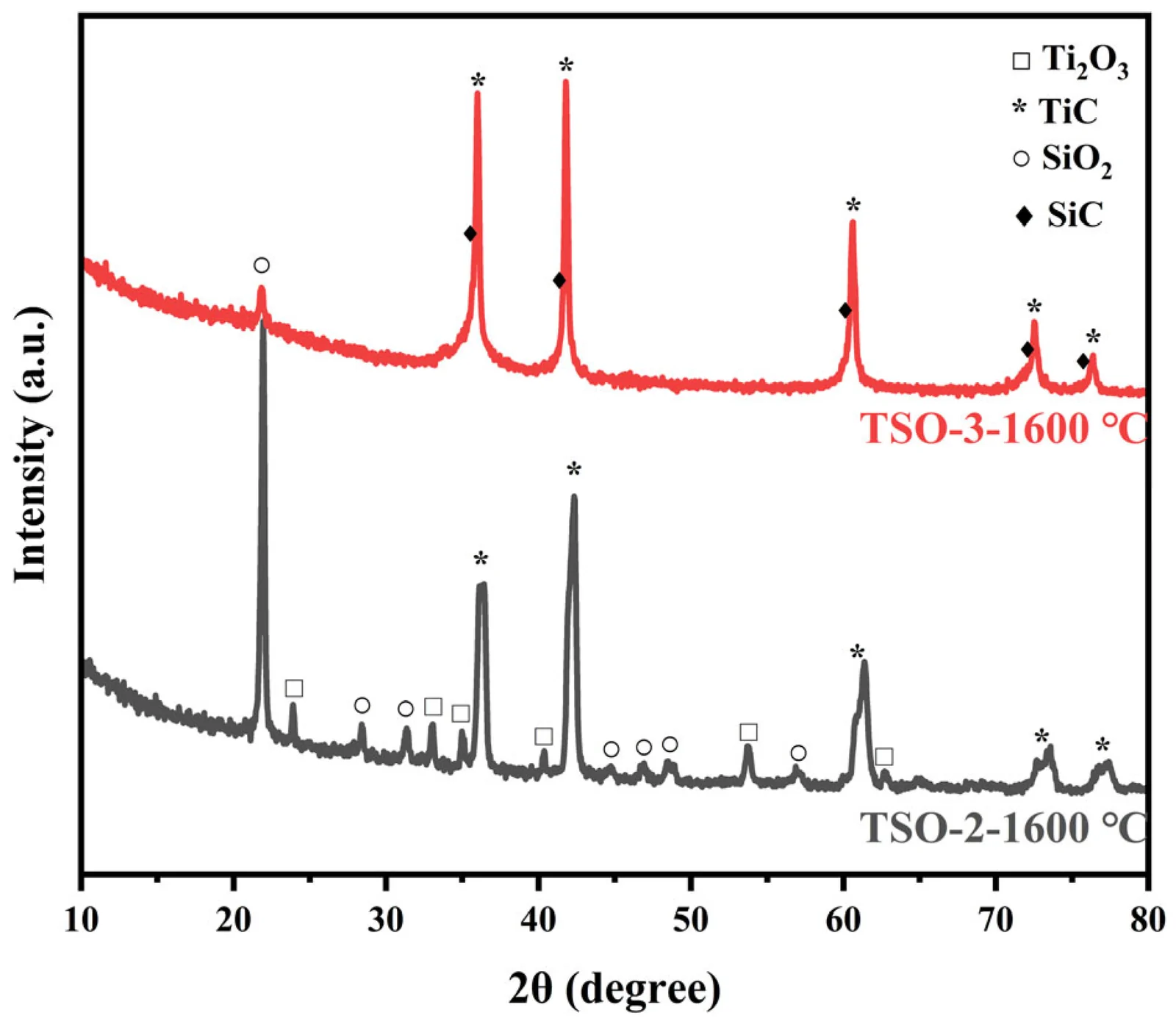
XRD patterns of TSO-2 and TSO-3 sintered at 1600 °C.

The absence of Ti_3_SiC_2_ in the TSO-1 product at 1600 °C is primarily attributed to the insufficient carbon source, which prevents the complete carbothermal reduction of SiO_2_ to SiO and CO, thereby interrupting the gas–solid reaction pathway due to the lack of essential gaseous reactants. Based on the above analysis, this section systematically investigates the effect of precursor composition on the phase composition. The binary TBT-LPCS system was retained, and the TBT/LPCS ratio was adjusted to increase the overall silicon and carbon contents. The effect of the TBT/LPCS ratio on the phase evolution was investigated. Two additional compositions were designed, with TBT/LPCS molar ratios of 3:4 and 3:10, denoted as TSO-2 and TSO-3, respectively. Figure 11 shows the XRD patterns of TSO-2 and TSO-3 sintered at 1600 °C.

For TSO-2, the products consist mainly of SiO_2_ and TiC, along with a small amount of Ti_2_O_3_. Compared with TSO-1, the Ti_3_O_5_ phase disappears and the Ti_2_O_3_ content decreases, indicating that the increased LPCS proportion provides more effective carbon and promotes the carbothermal reduction of titanium oxides. However, a considerable amount of SiO_2_ remains, indicating that the limited carbon source is still preferentially consumed in the carbothermal reduction of titanium oxides [30]. Notably, in comparison with standard TiC (PDF#32-1383), the diffraction peaks of TiC in TSO-2 are slightly shifted toward a larger 2θ angle, and a shoulder peak appears near the main peak. According to the literature, the covalent radius of Si (0.117 nm) is smaller than that of Ti (0.146 nm); therefore, in the Si-Ti-C system, Si atoms can diffuse into the lattice of TiC lattice at high temperature, partially substituting for Ti sites or occupying interstitial sites, which results in a decrease in lattice parameters and the shift in diffraction peaks to higher angle [31]. This peak shift indicates that, in TSO-2, although SiO_2_ is not substantially reduced, some Si atoms have migrated from the SiO_2_ or SiO_x_C_y_ matrix and dissolved in the TiC lattice to form the Ti(Si)C solid solution. Nevertheless, the effective carbon in the TSO-2 system is sufficient to reduce the titanium oxides to the TiC stage, but it cannot further drive the carbothermal reduction of SiO_2_ to SiO. Consequently, the reaction terminates in the coexistence of TiC and SiO_2_, and Ti_3_SiC_2_ cannot be formed. For TSO-3, SiC (PDF#73-1708) and TiC become the major phases, with a small amount of residual SiO_2_. The disappearance of titanium oxides indicates that the carbon content has exceeded the threshold required for the carbothermal reduction of titanium oxides. The appearance of the SiC phase is attributed to the pyrolysis of LPCS fractions that were not directly crosslinked with TBT.

**Figure 12:**
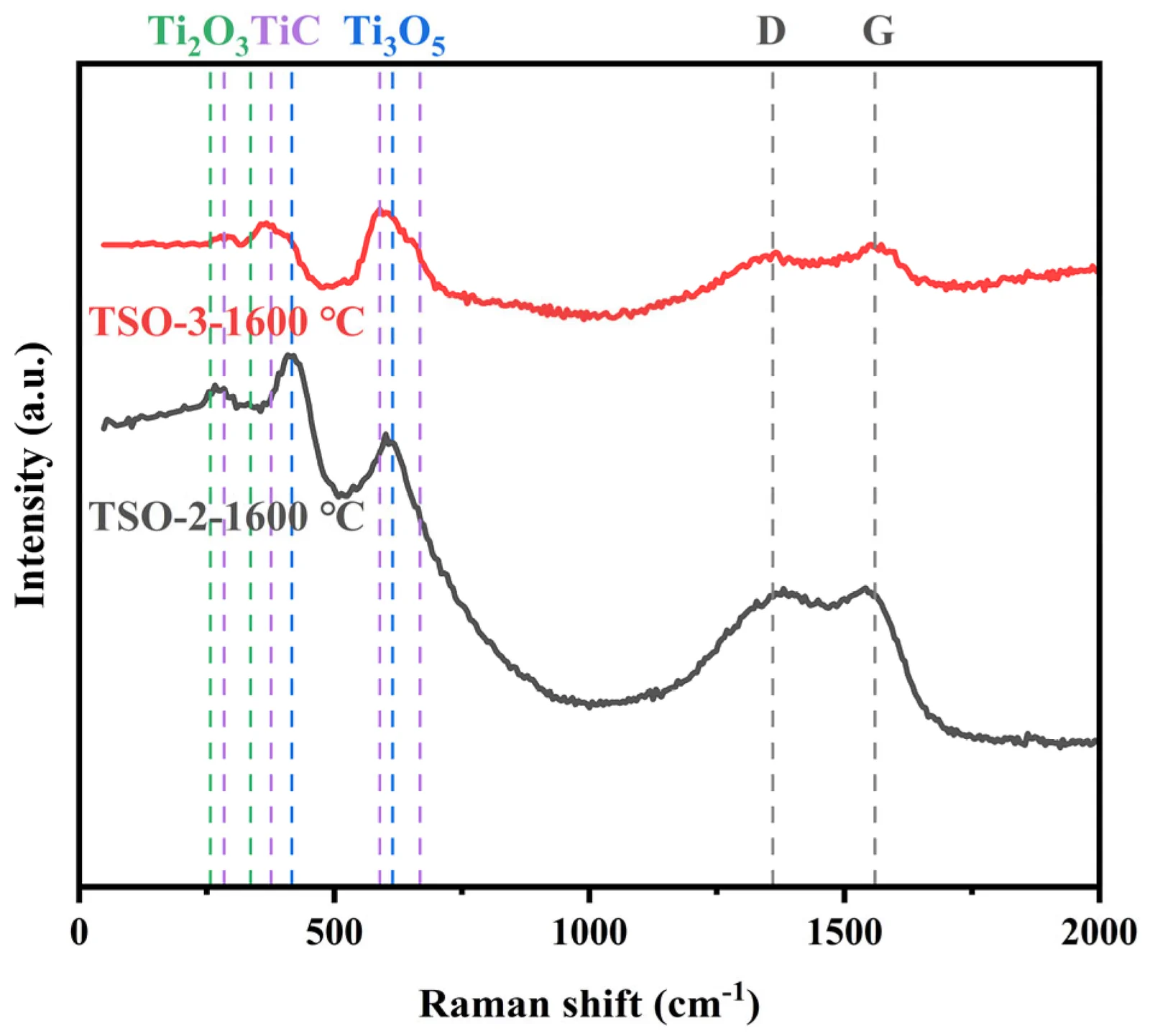
Raman spectra of TSO-2 and TSO-3 sintered at 1600 °C.

Figure 12 shows the Raman spectra of TSO-2 and TSO-3 sintered at 1600 °C. In TSO-2 and TSO-3, signal peaks corresponding to Ti_3_O_5_, Ti_2_O_3_, TiC, and free carbon are present, indicating the existence of a small amount of unreacted Ti_3_O_5_ in the system. The signal peak of SiC was not found in TSO-3, which may be because the SiC phase was obtained by pyrolysis of LPCS which was not directly cross-linked with TBT, and the free carbon formed a three-dimensional network around the SiC grains to cover the Si-C bond signal. Compared with TSO-1, the proportion of LPCS in TSO-2 increased, and the total carbon content of the system increased, so the I_D_/I_G_ increased significantly from 0.8 to 1.2. When the proportion of LPCS is further increased (TSO-3), a large amount of carbon in the system is used to construct the C-Si bond of SiC, causing I_D_/I_G_ ratio to decrease from 1.2 to 1.0.

The above compositional regulation reveals the underlying reason why Ti_3_SiC_2_ is difficult to form during the carbothermal reduction reaction of the Ti-Si-C-O precursor: even if the silicon and carbon contents of the system are increased, the carbon source generated during pyrolysis is preferentially consumed by the carbothermal reduction of titanium oxides. This carbothermal reduction of titanium oxides is thermodynamically more competitive than that of SiO_2_, which causes the system to bypass the reaction between Ti_2_O_3_ and SiO/CO gases.

## 4. Conclusions

The carbothermal reduction of fully liquid Ti-Si-C-O precursors was systematically investigated, with particular emphasis on the effects of binary composition adjustment on phase evolution. The main conclusions are as follows.

(1) The pyrolysis of the TSO-1 undergoes four stages: dehydration condensation (RT~200 °C), alkoxy removal (200~400 °C), inorganic conversion (400~800 °C), and carbothermal reduction (>800 °C). After sintering at 1400 °C and 1600 °C, the products are Ti_3_O_5_, SiO_2_ and TiC, Ti_3_O_5_, Ti_2_O_3_, SiO_2_ and TiC, respectively. However, Ti_3_SiC_2_ is not detected at either temperature. This is attributed to the large amount of escape of organic carbon during the pyrolysis of TSO-1, which leads to the insufficient total amount of effective carbon in the system, while the non-uniform distribution of residual carbon and the partitioned carbothermal reduction path further inhibit the gas–solid reaction path of Ti_3_SiC_2_.

(2) Increasing the LPCS ratio (TSO-2, TSO-3) promotes titanium oxide reduction to TiC, with partial Si atoms dissolving into the TiC lattice to form a Ti(Si)C solid solution. Nevertheless, the carbon source is preferentially consumed by the thermodynamically more favorable carbothermal reduction of titanium oxides, so SiO_2_ remains largely unreduced even when total Si and C contents are increased.

(3) The carbothermal reduction of Ti-Si-C-O precursors for Ti_3_SiC_2_ synthesis faces dual constraints: thermodynamically, titanium oxides outcompete SiO_2_ for carbon; kinetically, the gaseous escape of effective carbon and its microscale non-uniform distribution further impair the carbothermal reduction of SiO_2_. These combined factors prevent the gas–solid reaction from establishing, rendering Ti_3_SiC_2_ unattainable.

Our work still has the following limitations.

(1) This work focuses on the TBT-LPCS binary system and does not introduce external carbon sources to explore the effect of external carbon sources on the carbothermal reduction process.

(2) The pyrolysis experiment was conducted under a continuous argon gas flow. The gas flow inevitably carried away the SiO and CO gases required for the gas–solid reaction pathway. The influence of gas partial pressure on the Equations (4)–(6) was also not systematically quantified.

Based on the above analysis, the following strategies can improve the formation of Ti_3_SiC_2_: introducing external carbon sources such as phenolic resin to compensate for carbon loss; synthesizing new Ti-Si-C-O precursors with tailored elemental compositions; and adjusting the pyrolysis atmosphere and gas flow rate to promote the retention of SiO and CO.

## Figures and Tables

**Table 1:** The elemental composition and empirical formula of LPCS.

Sample	Si (wt%)	C (wt%)	H (wt%)	O (wt%)	Empirical Formula
LPCS	56.69	34.83	8.14	0.24	SiC_1.44_H_4.00_O_0.007_

**Table 2:** The denotations and composition of LPCS and TBT mixtures.

Samples	TBT:LPCS (Molar)	TBT:LPCS (Mass)	Ti:Si:O:C (Molar)
TSO-1	3:2	10:1	3:2:12:50.88
TSO-2	3:4	5:1	3:4:12:53.76
TSO-3	3:10	2:1	3:10:12:62.4

**Table 3:** The mass-to-charge ratio of the released fragments during pyrolysis and their corresponding functional groups.

*m*/*z*	2	16	18	28	44
functional groups	H_2_	CH_4_ or O^+^	H_2_O	C_2_H_4_ or CO	SiO

**Table 4:** Wavenumbers of common functional groups.

Wavenumber (cm^−1^)	Functional Group
650–750	Ti−O vibration
798, 1041, 1093	Ti-O-C vibration
970	Si-O-Ti vibration
1300–1500	C-H deformation vibration in CH_x_
1635	physically adsorbed water deformation vibration
2800–3000	C-H stretching vibration in CH_x_

**Table 5:** Phase mass percentages of TSO-1 pyrolyzed products.

Temperature	Ti_3_O_5_	Ti_2_O_3_	TiC	SiO_2_
1400 °C	50.8	—	10.5	38.7
1600 °C	10.6	26.6	21.7	41.1

## Data Availability

The original contributions presented in this study are included in the article. Further inquiries can be directed to the corresponding authors.
